# Outcomes of acute meningitis according to immunosuppression status: 15-year retrospective cohort

**DOI:** 10.1371/journal.pone.0344150

**Published:** 2026-03-24

**Authors:** Carla Marina Román-Montes, Valeria Alejandra Pérez-López, Nayeli Esmeralda Avalos-Celis, Aranza Castillejos-Gracia, Alfredo Ponce de León, Guillermo Arturo Guaracha-Basañez

**Affiliations:** 1 Infectious Diseases Department, Instituto Nacional de Ciencias Médicas y Nutrición Salvador Zubirán, Mexico City, Mexico; 2 Medicine School, Universidad Juárez Autónoma de Tabasco, Tabasco, Mexico; 3 Immunology and Rheumatology Department, Instituto Nacional de Ciencias Médicas y Nutrición Salvador Zubirán, Mexico City, Mexico; Cleveland Clinic Abu Dhabi, UNITED ARAB EMIRATES

## Abstract

Acute meningitis remains a primary global health concern. Immunosuppressed patients have risks due to atypical clinical presentations and a broader range of causative pathogens. We aimed to describe the outcomes and clinical features of acute meningitis according to immunosuppression status. We performed a retrospective cohort study of adults with acute meningitis from January 2009 to December 2023. Patients with postsurgical meningitis were excluded. Outcomes and demographic, clinical, and laboratory features were compared using non-parametric statistical tests, and mortality was analyzed using multivariate logistic regression. Among 189 patients, 96 (51%) were immunosuppressed. The median age was lower in immunosuppressed patients (36 vs. 50 years, p < 0.01). There were no differences in symptoms; the classical triad was present in only 21% vs. 19%. Immunosuppressed patients had lower CSF glucose levels (59% vs. 39%, p = 0.004). Overall mortality was 20%, with no significant difference by immune status. Independent predictors of death included age over 50 years (OR 2.9), altered mental status (OR 4.7), and bacterial meningitis (OR 2.3). Acute meningitis in immunosuppressed hosts shows attenuated inflammatory CSF profiles and a broader etiologic spectrum. Immunosuppression was not independently associated with in-hospital mortality.

## Introduction

Acute meningitis remains a major global health problem, causing significant morbidity and mortality despite advances in prevention and treatment [1,2]. Globally, it accounts for an estimated 2.8 million cases and over 300,000 deaths each year, with the burden highest in low- and middle-income countries [3]. Bacterial meningitis is particularly severe, as it can rapidly progress to neurologic damage or death if not promptly treated [4].

In immunosuppressed populations, such as individuals with HIV/AIDS, solid-organ transplant recipients, patients on prolonged corticosteroids or immunosuppressive therapies for rheumatic disease, and those with hematologic malignancies, the risk of meningitis is markedly increased [5]. In these hosts, the etiologic spectrum expands beyond typical pathogens to include opportunistic organisms such as *Listeria monocytogenes*, *Pseudomonas aeruginosa*, *Cryptococcus neoformans*, and *Mycobacterium tuberculosis* [6,7].

Data from low- and middle-income countries, especially among patients with a high burden of rheumatic diseases and other non-HIV immunosuppression, remain scarce. In Latin America, most available studies on acute meningitis focus on HIV infection or community-acquired disease in the general population [8–10], with limited information on how immunosuppression influences clinical presentation, etiologic spectrum, and outcomes.

To address this gap, we conducted a 15-year retrospective analysis at a tertiary referral center in Latin America to compare the characteristics and outcomes of acute meningitis according to immunosuppression status and to identify independent predictors of in-hospital mortality.

## Materials and methods

We conducted a retrospective cohort analysis of acute meningitis cases attended from January 2009 to December 2023 at a tertiary care center in Mexico City, examining outcomes according to immunosuppression status.

### Population

Patients aged 18 years or older who met the definition of acute meningitis were included. Cases associated with neurosurgical procedures were excluded to avoid bias. Data were extracted from electronic medical records between August and November 2025. Demographic, clinical, and laboratory variables were collected from electronic files.

### Definitions

Acute meningitis was defined as an acute central nervous system infectious syndrome characterized by headache [11,12] and/or fever plus at least one meningeal or neurological sign (neck stiffness, photophobia, seizures, or altered mental status [AMS]), together with cerebrospinal fluid (CSF) findings compatible with meningeal inflammation (CSF pleocytosis, elevated CSF protein, and low CSF glucose).

Low CSF glucose was defined as a CSF glucose level <45 mg/dL or a CSF-to-serum glucose ratio ≤0.5. Elevated CSF protein was defined as a CSF protein level >45 mg/dL, and CSF pleocytosis was defined as a CSF leukocyte count >5 cells/mm^3^.

Aseptic meningitis was defined as acute meningitis without microbiologic or etiologic evidence.

Tuberculous and cryptococcal meningitis were included when presenting within 14 days of symptom onset and with microbiological confirmation, to reflect the real-world diagnostic spectrum at our center. A sensitivity analysis excluding *Mycobacterium tuberculosis* and *Cryptococcus* spp. cases was performed [11,12].

Immunosuppression was defined a priori as the presence of at least one of the following at meningitis onset:

(1) receipt of systemic corticosteroids equivalent to ≥10 mg/day of prednisone for ≥4 consecutive weeks; (2) treatment within the previous 6 months with conventional synthetic or biologic disease-modifying antirheumatic drugs (DMARDs), calcineurin inhibitors, antimetabolites (e.g., azathioprine, mycophenolate mofetil), alkylating agents, tyrosine kinase inhibitors, or T- or B-cell–depleting agents (e.g., rituximab, cyclophosphamide); (3) recent cytotoxic chemotherapy for solid or hematologic malignancy; (4) solid organ or hematopoietic stem cell transplantation; (5) advanced HIV infection (CD4 cell count <200 cells/μL or any AIDS-defining condition); (6) documented inborn error of immunity; (7) severe neutropenia <0.5 × 10^9^ neutrophils/L lasting >10 days temporally related to meningitis onset.

Comorbidities such as type 2 diabetes mellitus, obesity, chronic liver disease, chronic kidney disease, and chronic lung disease were recorded but did not meet the immunosuppression definition unless one of the above criteria was present [13].

### Statistical analysis

Categorical variables are presented as frequencies and percentages, and continuous variables as medians with interquartile ranges (IQR). Distribution was assessed using the Shapiro–Wilk test.

Comparisons between immunosuppressed and non-immunosuppressed patients were performed using the $\chi^{2}$ or Fisher’s exact test for categorical variables and the Mann–Whitney U or Kruskal–Wallis test for continuous variables.

The primary outcome was in-hospital mortality. Candidate predictors were first evaluated in bivariable analyses. Variables associated with mortality at *p* < 0.20 and/or considered clinically relevant were entered into a multivariable logistic regression model.

Model performance was assessed using discrimination (area under the receiver operating characteristic curve) and calibration (goodness-of-fit test). Sensitivity and specificity were not emphasized because the model was not developed for binary risk classification.

All analyses were conducted using Stata version 14.0 (StataCorp, College Station, TX, USA). A two-sided *p* < 0.05 was considered statistically significant.

## Results

A total of 189 cases of acute meningitis were identified. Most patients were women (105/189, 55.5%), with a median age of 41 years (IQR 30–57). Immunosuppression was present in 96 (51%) patients. There were no sex differences between groups, and the median age was lower in the immunosuppressed group (36 [IQR 27–50.5] vs. 50 [IQR 33–64] years, *p* < 0.01).

Demographic characteristics and comorbidities according to immunosuppression status are summarized in Table 1.

**Table 1:** Demographic and comorbidities of patients with acute meningitis according to immunosuppression status.

Characteristic	Overall	Immunosuppressed	Non-immunosuppressed
	(N = 189)	(n = 96)	(n = 93)
Female sex	105 (55.5%)	52 (54%)	53 (57%)
Age, median (IQR)	41 (30–57)	36 (27–50.5)	50 (33–64)
Age > 50 years	70 (37%)	24 (25%)	46 (49.5%)
Obesity	13 (7%)	1 (1%)	12 (13%)
Rheumatic disease	60 (32%)	49 (51%)	11 (12%)
HIV infection	37 (20%)	30 (31%)	7 (7.5%)
Hematologic malignancy	10 (5%)	9 (9%)	1 (1%)
Type 2 diabetes	41 (22%)	13 (13.5%)	28 (30%)
Chronic kidney disease	19 (10%)	10 (10%)	9 (10%)
Chronic liver disease	16 (8.5%)	4 (4%)	12 (13%)
Solid neoplasm	3 (2%)	2 (2%)	1 (1%)
Solid organ transplant	10 (5%)	9 (9%)	1 (1%)

IQR: interquartile range. HIV: human immunodeficiency virus. Data are presented as n (%) unless otherwise indicated.

Among immunosuppressed patients, 70/96 (73%) had pharmacological immunosuppression. Of these, 60/70 (86%) received prednisone (median dose 20 mg, IQR 5–50). The most frequently used DMARDs were methotrexate (24%), azathioprine (21%), mycophenolate (19%), tacrolimus (10%), and cyclophosphamide (10%). Advanced HIV infection accounted for 20/96 (31%) cases; the median CD4 count was 25 cells/μL (IQR 0–125).

### Clinical characteristics

The median duration of symptoms prior to emergency department presentation was 3 days (IQR 1–7), with no significant difference between groups (*p* = 0.15).

There were no significant differences in clinical manifestations between immunosuppressed and non-immunosuppressed patients (Table 2). The most frequent symptoms were headache, fever, and altered mental status. The classical triad (fever, neck rigidity, and altered mental status) was present in 21% vs. 19% of patients (*p* = 0.80).

**Table 2:** Clinical and diagnostic features in patients with acute meningitis according to immunosuppression status.

	Overall (N = 189)	Immunosuppressed (n = 96)	Nonimmunosuppressed (n = 93)
**Clinical features**			
Headache	140 (74%)	75 (78%)	65 (70%)
Fever	138 (73%)	70 (73%)	68 (73%)
Altered mental status	116 (61%)	60 (62.5%)	56 (60%)
Vomiting	64 (34%)	38 (40%)	26 (28%)
Neck pain	43 (23%)	20 (21%)	23 (25%)
Seizures	42 (22%)	23 (24%)	19 (20%)
Nuchal rigidity	77 (41%)	34 (35%)	43 (46%)
Photophobia	27 (14%)	14 (15%)	13 (14%)
GCS < 15	85 (45%)	44 (46%)	41 (44%)
Focal neurological signs	86 (45.5%)	46 (48%)	40 (43%)
Classical triad	38 (20%)	20 (21%)	18 (19%)
Any meningeal sign	33 (17.5%)	13 (13.5%)	20 (21.5%)
**Laboratory findings**			
Leukocyte count (cells/mm^3^), median (IQR)	8400 (5100–13100)	6200 (4500–10050)	10500 (7100–14500)
Leukocytosis	65 (34%)	21 (22%)	44 (47%)
Hyponatremia	85 (45%)	43 (45%)	42 (45%)
Serum glucose (mg/dL), median (IQR)	112 (95–138)	108.5 (94–125)	115 (98–149)
**CSF findings**			
CSF glucose (mg/dL), median (IQR)	45 (20–60)	40 (16–54)	52 (24–68.7)
Low CSF glucose	93 (49%)	57 (59%)	36 (39%)
CSF protein (mg/dL), median (IQR)	112.5 (50.5–233.5)	117.6 (52.5–262.3)	110.1 (50–200)
Elevated CSF protein	155 (82%)	80 (83%)	75 (81%)
CSF leukocytes (cells/mm^3^), median (IQR)	55 (7–205)	35 (5–150)	96 (10–224)
CSF pleocytosis	144 (74%)	69 (72%)	75 (81%)
Positive CSF culture	92 (49%)	50 (52%)	42 (45%)
Positive CSF CrAg	16 (9%)	14 (16%)	2 (3%)
Positive CSF tuberculosis PCR	10 (8%)	9 (14%)	1 (1.5%)

GCS: Glasgow Coma Scale. CSF: cerebrospinal fluid. CrAg: cryptococcal antigen. PCR: polymerase chain reaction. IQR: interquartile range. Data are presented as n (%) unless otherwise indicated.

### Laboratory and CSF findings

All patients underwent lumbar puncture. Immunosuppressed patients had significantly lower CSF glucose levels (median 40 vs. 52 mg/dL, *p* = 0.02). There was no statistically significant difference in CSF leukocyte count (median 35 vs. 96 cells/mm^3^, *p* = 0.06) nor in the proportion with pleocytosis or elevated CSF protein.

Etiologic diagnosis was more frequently established among immunosuppressed patients (76/96, 79% vs. 54/93, 58%; *p* = 0.002). The proportion of aseptic meningitis was lower in immunosuppressed patients (21% vs. 42%; *p* = 0.002).

**Fig 1:**
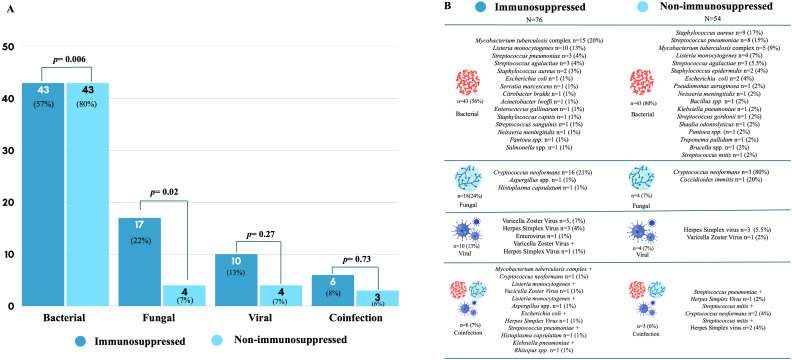
Etiology of acute meningitis according to immunosuppression status.

The most common etiologic category in immunosuppressed patients was *Mycobacterium tuberculosis* complex, whereas *Streptococcus pneumoniae* predominated among non-immunosuppressed individuals. The distribution of etiologic agents is shown in Fig 1.

CSF culture was positive in 92 (49%) patients, without differences by immunosuppression status (*p* = 0.34). Blood cultures were obtained in 152 (80%) patients and were positive in 42 (28%), with no significant differences between groups (*p* = 0.16).

### Mortality analysis

Overall in-hospital mortality was 38/189 (20%), without significant differences according to immunosuppression status (18% vs. 23%; *p* = 0.40). In multivariable logistic regression analysis, independent predictors of mortality included:

- Age ≥50 years (OR 2.9, 95% CI 1.31–6.37, *p* = 0.01)
- Altered mental status (OR 4.7, 95% CI 1.70–12.98, *p* = 0.003)
- Bacterial meningitis (OR 2.3, 95% CI 1.01–5.1, *p* = 0.04)

Immunosuppression was not independently associated with mortality (OR 0.73, 95% CI 0.36–2.6; *p*=0.40).

**Fig 2:**
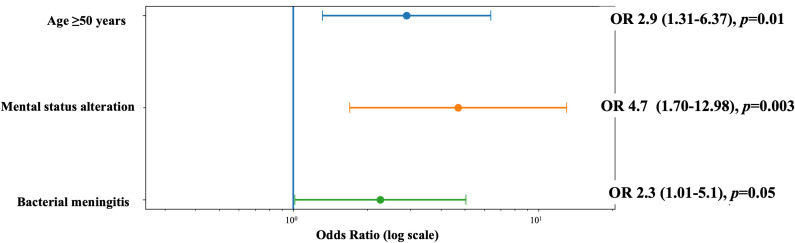
Multivariable logistic regression model for in-hospital mortality.

The final multivariable model demonstrated good discrimination (AUC = 0.75) and adequate calibration (Pearson goodness-of-fit *p* = 0.52). The forest plot of the regression model is shown in Fig 2.

## Discussion

This study analyzes 15 years of acute meningitis at a tertiary referral center, with more than half of cases occurring in immunosuppressed patients, underscoring the importance of infections in this vulnerable population. Despite demographic and laboratory differences, mortality rates were similar between immunosuppressed and non-immunosuppressed patients, suggesting that immunosuppression alone may not be the primary determinant of short-term mortality.

Consistent with previous reports, only 20% of patients presented with the classical triad of meningitis, particularly among immunosuppressed individuals [5]. Clinical manifestations such as headache and fever were similar regardless of immune status. Attenuated inflammatory responses likely explain the lower CSF leukocyte counts and glucose levels observed in immunosuppressed patients [14,15]. These findings highlight the importance of maintaining a high index of suspicion and performing early lumbar puncture in immunocompromised patients presenting with nonspecific neurological symptoms, even in the absence of classic meningeal signs.

An etiologic diagnosis was more frequently established in immunosuppressed patients. Opportunistic infections in this group may yield higher microbiological diagnostic rates due to routine use of antigen testing, PCR-based methods, and fungal cultures. Previous studies have reported delayed diagnosis and higher false-negative rates in non-immunosuppressed populations [16]. In adults with community-acquired meningitis, low CSF glucose has been associated with increased likelihood of microbiologic confirmation and treatable etiologies [17], findings that are consistent with our results.

The etiologic spectrum observed in our cohort reflects regional epidemiology. *Mycobacterium tuberculosis* represented a significant proportion of cases among immunosuppressed patients, consistent with the burden of tuberculosis in Mexico [16]. Opportunistic pathogens such as *Cryptococcus neoformans* and *Listeria monocytogenes* were also prominent in this group, as previously described in HIV and transplant populations [6]. In contrast, *Streptococcus pneumoniae* remained the leading pathogen among non-immunosuppressed individuals.

In multivariable analysis, age ≥50 years, altered mental status, and bacterial etiology were independently associated with mortality. These findings align with prior studies in adult bacterial meningitis [1,18–20]. Older age and decreased level of consciousness at presentation have consistently been identified as strong predictors of unfavorable outcomes across different meningitis etiologies, including cryptococcal meningitis [21].

The absence of an independent association between immunosuppression and mortality should be interpreted cautiously. Younger age among immunosuppressed patients, higher microbiologic diagnostic yield, and confounding by etiologic distribution may partially explain this finding. Residual confounding inherent to retrospective analyses cannot be excluded.

This study has limitations. Its retrospective, single-center design may limit generalizability. Long-term neurological outcomes beyond hospital discharge were not evaluated. Furthermore, the inclusion of microbiologically confirmed tuberculous and cryptococcal meningitis presenting within 14 days may affect comparability with cohorts of acute bacterial or viral meningitis, although a sensitivity analysis was performed. A major strength is the 15-year follow-up period.

## Conclusions

In this 15-year retrospective cohort, immunosuppressed patients with acute meningitis exhibited attenuated cerebrospinal fluid inflammatory profiles and a broader etiologic spectrum compared with non-immunosuppressed individuals. However, immunosuppression was not independently associated with in-hospital mortality after adjustment for age, neurologic severity, and etiology.

Age ≥50 years, altered mental status at presentation, and bacterial etiology were independent predictors of death. These findings underscore the importance of early recognition, prompt diagnostic evaluation, and appropriate empiric antimicrobial therapy in all patients with suspected acute meningitis, particularly in those with impaired immune function.

## Supporting information

S1 Fig Sensitivity analysis excluding tuberculosis and fungal meningitis. (TIFF)

S1 Table Additional multivariable model excluding fungal cases. (PDF)
