# Supplementary figures and images for: Outcomes of acute meningitis according to immunosuppression status: 15-year retrospective cohort
Source: PLoS One. 2026 Mar 24;21(3):e0344150. doi: 10.1371/journal.pone.0344150 (PMC13012731; doi:10.1371/journal.pone.0344150)

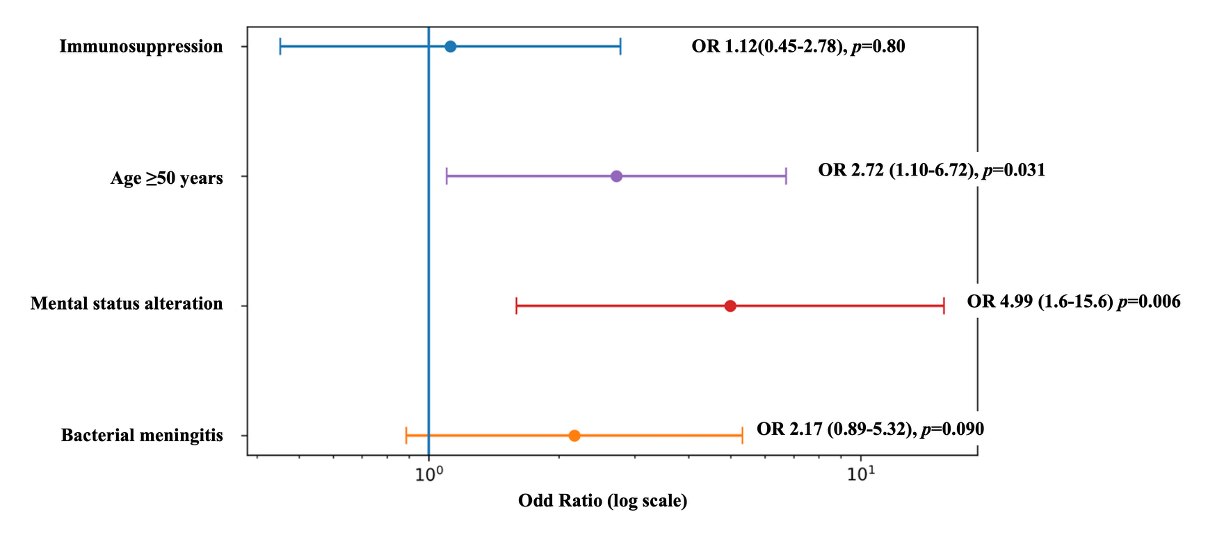

Supplement: S1 Fig — (TIFF) [file pone.0344150.s001.tiff]
