# Supplementary material for: Outcomes of acute meningitis according to immunosuppression status: 15-year retrospective cohort
Source: PLoS One. 2026 Mar 24;21(3):e0344150. doi: 10.1371/journal.pone.0344150 (PMC13012731; doi:10.1371/journal.pone.0344150)
Supplement: S1 Table — (PDF) [file pone.0344150.s002.pdf]

Table S1. Demographic, comorbidities, and meningitis features of acute meningitis patients by immunosuppression status, excluding tuberculosis and fungal meningitis.

**Characteristics**	**Overall N=149(%)**	**Immunosuppressed n=65(%)**	**Non immunosuppressed n=84(%)**	***p*-value**
Female sex	90 (60)	43 (66)	47 (56)	0.20
Age, median (IQR), years	44 (30-59)	36 (27-52)	50 (32.5-65)	0.002
Age >50 years	61(41)	20 (31)	41 (49)	0.02
Rheumatic disease	51 (34)	40 (61.5)	11(13)	<0.001
HIV infection	17 (11)	10(15)	7(8)	0.17
Hematologic malignancy	10 (7)	9(14)	1(1)	0.003
Type 2 Diabetes	37 (25)	11(17)	26(31)	0.05
Chronic liver disease	13 (9)	4(6)	9(11)	0.39
Solid organ transplant	9(6)	8(12)	1(1)	0.01
		Clinical features		
Headache	112(75)	52(80)	60(71)	0.23
Fever	109(73)	50(77)	59(70)	0.36
Altered mental status	86(58)	36(55)	50(59)	0.61
Vomiting	47(31.5)	26(40)	21(25)	0.05
Classical meningitis triad	27(18)	11(17)	16(19)	0.73
		Diagnostic		
Leukocytosis	62(42)	22(34)	40(48)	0.09
Positive Blood cultures	35/118(30)	18/48(37.5)	17/70 (24)	0.12
Abnormal head CT	43/139 (31)	16/64(25)	27/75 (36)	0.16
Etiologic diagnosis	90(60)	45(69)	45(54)	0.05
		CSF characteristics		
CSF *pH**	7.5(7.39-7.64)	7.5(7.38-7.64)	7.49(7.4-7.62)	0.93
Low CSF glucose	64(43)	34(52)	30(36)	0.04
Elevated CSF protein	118 (79)	51 (78.5)	67 (80)	0.85
CSF pleocytosis	118(79)	48(74)	70(83)	0.16
CSF positive culture	62 (42)	30(46)	32(38)	0.38

Abbreviations: CSF, cerebrospinal fluid; CT, computed tomography; HIV, human immunodeficiency virus; IQR, interquartile range.

Table S2. Demographic and Clinical Features of meningitis by etiology.

	**Bacterial N=28(%)**	**Tuberculosis N=16(%)**	**Fungal N=17(%)**	**Viral N=10 (%)**	***p-value***
**Immunosuppressed n=71**					
Age, median (IQR)	36(23.5-52)	39(32-50)	32(28-38)	33.5(27-42)	0.44
Female sex<br>Male	22 (79)<br>6 (21)	6 (37.5)<br>10 (62.5)	4 (23.5)<br>13 (76.5)	7 (70)<br>3 (30)	0.001
Cause of immunosuppression					
Pharmacologic due rheumatic disease	20 (71)	7(44)	2(12)	5(50)	0.002
HIV advanced	1 (4)	8(50)	12(71)	2(20)	<0.01
Pharmacologic due transplant	2(7)	0	1(6)	3(30)	0.05
Other cause	5(18)	1(6)	2(12)	0	0.40
Prednisone use	26 (93)	5 (31)	5 (29)	6(60)	0.04
Meningitis characteristics					
Headache	21(75)	8(50)	17(100)	8(80)	0.01
Fever	23 (51)	10 (22)	12 (27)	6(60)	0.41
Altered mental status	15(54)	14(87.5)	10(59)	5(50)	0.09
CSF *pH**	7.48 (7.2-7.62)	7.46 (7.36-7.56)	7.45 (7.42-7.53)	7.7 (7.53-7-78)	0.02
Low CSF glucose	19(68)	13(81)	10(59)	3(30)	0.06
Elevated CSF protein	23(82)	16(100)	14(82)	8(80)	0.33
CSF pleocytosis	21 (75)	11(69)	11(65)	7(70)	0.90
Death at 12 weeks	6(21)	4(25)	3(1)	0	0.18
**Nonimmunosuppressed n=49**	Bacterial N=38(%)	Tuberculosis N=4(%)	Fungal N=4(%)	Viral N=3 (%)	*p-value*
Age, median (IQR)	36 (23.5-52)	39(32-50)	32(28-38)	33.5(27-42)	0.44
Female sex<br>Male sex	23(60.5)<br>15(39.5)	1(25)<br>3(75)	0<br>4(100)	1(33)<br>2(67)	0.18
Rheumatic disease	6(16)	0	1(25)	0	0.85
Type 2 diabetes	15(39.5)	0	2(50)	1(33)	0.50
Chronic liver disease	6(16)	1(25)	0	2(67)	0.10
Chronic kidney disease	5(13)	0	0	0	1.0
Meningitis characteristics					
Headache	23(60.5)	2(50)	3(75)	3(100)	0.49
Fever	28(74)	4(100)	2(50)	3(100)	0.29
Altered mental status	29(76)	3(75)	2(50)	1(33)	0.32
Classic triad	10(26)	0	0	1(33)	0.41
CSF *pH**	7.48 (7.32-7.7)	7.84 (7.67-7.9)	7.48 (7.18-7.68)	7.87 (7-8)	0.17
Low CSF glucose	19(50)	3(75)	1(25)	1(33)	0.51
Elevated CSF protein	33(87)	3(75)	3(75)	3(100)	0.73
CSF pleocytosis	31(82)	3(75)	4(100)	2(67)	0.69

Death at 12 weeks	10(26)	1(25)	0	2(67)	0.27

Abbreviations: CSF, cerebrospinal fluid; HIV, human immunodeficiency virus; IQR, interquartile range.

Figure 1S. Mortality analysis using logistic regression in acute meningitis patients, without tuberculosis and fungal meningitis. Forest plot of multivariable logistic regression excluding tuberculous and fungal cases, showing factors associated with 12-week mortality in patients with acute meningitis. Altered mental status and age ≥50 years were independently associated with increased mortality. Odds ratios with 95% confidence intervals and *p*-values are shown. The multivariable model demonstrated good discrimination (AUC = 0.76) and adequate calibration, with no lack of fit (Pearson test, p = 0.40).
